# The Gaseous Electronics Conference RF Reference Cell—An Introduction

**DOI:** 10.6028/jres.100.025

**Published:** 1995

**Authors:** J. K. Olthoff, K. E. Greenberg

**Affiliations:** National Institute of Standards and Technology, Gaithersburg, MD 20899-0001; University of New Mexico, Albuquerque, NM 87131

**Keywords:** Gaseous Electronics Conference, plasma reactor, radio frequency, reference cell

## Abstract

This paper provides an introduction to the Gaseous Electronics Conference (GEC) RF Reference Cell, and to the articles published in this Special Issue of the *Journal of Research of the National Institute of Standards and Technology*. A brief summary of the history and purpose of the Reference Cell concept is presented, and recent changes to the GEC Cell design are documented. The paper concludes with highlights of research performed on GEC Cells, and with an appendix of all known publications that present research performed on GEC Cells.

## 1. Introduction

One of the most important commercial applications of low-temperature glow discharges is the fabrication of microelectronic circuitry. Low temperature plasmas are indispensable for etching the fine features needed in large-scale device integration. These plasmas are also used to deposit materials, remove photoresist, and enhance oxidation. However, many plasma-based processes used to produce integrated circuits suffer from reliability problems. One process may work well for some time and then fail mysteriously, while another may yield certain results in one reactor, and completely different results in another. These types of problems cause decreased efficiency and increased costs for the semiconductor manufacturing industry. These same problems inhibit research studies of the physical processes occurring in these processing plasmas, due to the difficulty in comparing experimental results obtained on different reactors, and the difficulty in comparing experimental data with the results derived from theoretical models.

Discharge characteristics can be affected by the configuration of the electrodes and chamber, materials of construction, design of the power circuitry, location of the diagnostic probes, and various other parameters such as surface conditioning and gas impurities. While the basic effects of these variables are generally acknowledged, there has been little systematic research to determine their influence on the discharge. The presence of so many interrelated parameters that vary from apparatus to apparatus makes it difficult to isolate the processes that cause the plasma to change from experiment to experiment. Because of this, there has been considerable debate about the set of operating parameters that must be controlled and the precision that measurements must have in order to replicate experiments at different laboratories.

An attempt to formally address these problems at the 1988 Gaseous Electronics Conference (GEC) resulted in the design of the GEC RF Reference Cell: a parallel plate, capacitively-coupled, rf plasma reactor that, in principal, is suitable for studies of basic discharge phenomena, investigation of industrial-type plasmas, and theoretical modeling. The use of several of these chambers to perform similar experiments in different laboratories was proposed to aid in isolating the effects of reactor geometry from other experimental variables. By equipping ostensibly identical cells with a wide variety of diagnostic tools, such as voltage and current probes, lasers, Langmuir probes, and mass spectrometers, a comparison of measurements would eventually allow an assessment of the extent to which an rf discharge can be reliably defined and reproduced in the laboratory.

Many laboratories have since utilized GEC RF Reference Cells (or GEC Cells, for short) in their discharge or plasma processing research programs. The GEC Cell has also been used as a successful teaching tool in both graduate and undergraduate labs, primarily because of the combination of simplicity and diagnostic access [1]. To date, nearly 25 GEC Reference Cells are in use at 19 different industrial, academic, and government laboratories, with the related research resulting in the publication of over 75 articles and reports (see Appendix A). As the use of GEC Cells has expanded, it has become apparent that a review of the progress made in addressing the problems discussed above would be useful. It was therefore decided that, in conjunction with the hosting of the 1994 Gaseous Electronics Conference by the National Institute of Standards and Technology, a Special Issue of the *Journal of Research of the National Institute of Standards and Technology* would be dedicated to the review and analysis of research performed on GEC RF Reference Cells. This Special Issue, in addition to being a review of techniques, diagnostics, and models applied to the GEC Cell, is also intended to serve as a “users’ guide” and reference source for individuals working with GEC RF Reference Cells.

This Special Issue contains twelve articles, including this introductory paper, that cover most of the research performed on GEC Cells. Six of the articles describe various diagnostic techniques that have been used with the GEC Cells, including electrical measurements, optical emission, laser-induced fluorescence, mass spectrometry with ion energy analysis, microwave interferometry, and Langmuir probes. The remaining articles discuss one- and two-dimensional modeling of the GEC Cell, the development of a high density inductively coupled plasma source, semiconductor etching performed in GEC Cells, and the investigation of particulate formation. The papers are primarily meant to be a review of how these experimental or modeling studies were performed on GEC Cells, with an emphasis on the comparison of results from different laboratories in order to address the question of plasma reproducibility.

This introductory article begins with a brief history of the development of the GEC Cell concept and design, along with a more detailed discussion of the overall purpose for its existence. The article continues with a description of the basic GEC Cell design and operation, including a discussion of various modifications that have been made to GEC Cells in various laboratories, and concludes with highlights of some of the major accomplishments of the effort.

## 2. Purpose and History of the GEC RF Reference Cell

As mentioned in the Introduction, experimental data obtained for low-temperature glow discharges have often been difficult to compare when obtained by different research groups using different plasma reactors. Researchers have typically picked a discharge geometry to accommodate particular diagnostic techniques, and since the discharge geometry strongly affects the plasma properties, it has been unclear whether meaningful comparisons could be made between data obtained in different plasma systems. Furthermore, it has been difficult to compare results from fundamental discharge models with the wide variety of measurements made in rf systems having different geometries. It seems that it has always been possible to find one set of experimental results that would support the results of a particular model.

In order to address the problems of data comparison and model validation for rf discharges, a workshop entitled “Design, Calibration, and Modeling of RF Plasma Processing Systems” was held at the 1988 Gaseous Electronics Conference (Minneapolis, MN, Oct. 18–21). During the workshop, it was generally agreed that development of a “reference cell” could help to facilitate comparisons of experimental and theoretical data. Furthermore, it was agreed that development of a reference cell system had to include specification of a common discharge geometry and the specification of a “minimum diagnostic tool set.” The diagnostics would be used to determine whether seemingly identical reference cells operated similarly. It was hoped that by specifying a common geometry and a set of diagnostics it would be possible to insure that plasmas generated under similar conditions (pressure, power, flow) in reference cells of similar design would have similar properties. It was further hoped that a variety of different experimental measurements would be made in these systems to provide a database that could be used for validation of rf discharge models.

The 1988 GEC workshop resulted in the establishment of an ad hoc committee comprised of both researchers and technologists from universities, national laboratories, and industry. The committee was assigned the task of developing a preliminary design for a cost-effective reference cell system that: 1) could be easily replicated; 2) would be able to accommodate a variety of diagnostics; and 3) would be technologically relevant. Regarding the goal of technological relevance, it was further specified that the reference cell design was to be related to geometries used by the semiconductor industry, and that the reference cell was to be capable of generating discharges in reactive or “etching” gases used for semiconductor processing. However, it was also considered essential that the design be as “simple” as possible in order to facilitate modeling efforts. A preliminary design was specified, and in March 1989, was finalized during a meeting of the ad hoc committee and additional representatives from universities, national laboratories, industry, and government. This reactor was unofficially named the “GEC RF Reference Cell,” and six different research groups^1^ agreed to procure GEC Cell systems and use them to make diagnostic measurements.

At the 1989 Gaseous Electronics Conference (Palo Alto, CA, Oct. 17–20), a workshop was held to discuss the diagnostic tool set that would be used to characterize GEC Cell operation. Different workshop participants advocated a variety of different measurement techniques for inclusion in the tool set. Some of the proposed diagnostics were voltage and current, Langmuir probe, spatially-resolved optical emission, mass spectrometry, and microwave (electron density) measurements. The participants also expressed a desire that the chosen diagnostics be easy to implement, be easy to interpret, and be relatively inexpensive.^2^

As a result of the workshop and subsequent meetings, it was decided that measurements of voltage and current waveforms (including dc bias) would be easiest to implement by all groups and would yield the least ambiguous results. It was felt that discharges having identical voltage and current waveforms for a particular set of input conditions (applied voltage, pressure, and gas flow) would probably have similar plasma properties, although this premise had yet to be proven systematically. Therefore, in order to compare operation of the different GEC Cells, an agreed upon set of rf peak-to-peak voltages was used to generate discharges in ultrahigh-purity argon, and the resulting voltage and current wave forms were recorded. The data used for comparison consisted of the amplitudes and phases of the first five Fourier components of the voltage and current, the self-bias voltage, and the calculated power dissipated in the discharge. These data sets were gathered over a range of pressures from 13.3 Pa to 133 Pa.

There were a number of reasons why an inert gas was chosen even though a major purpose of the GEC Cell is to study discharges in reactive gases of interest to industry. First, a “nonreactive” gas would provide a more controlled experiment for comparing the characteristics of different GEC Cells. Second, a “nonreactive” gas would serve as a benchmark for the behavior of a “clean” GEC Cell. Third, the initial data could be used to help validate theoretical models of the rf discharge. Discharges generated using inert gases are among the easiest to model since the plasma chemistry is simplified and more fundamental data (e.g., cross sections) are available for these gases. Once accurate models of inert gas discharges have been “validated” by comparison with measured data using the GEC Cell, they can then be extended to the more complex systems used in industry. Models should eventually reproduce the results of all observations, e.g., measured voltage and current characteristics, ion kinetic energy distributions, electron densities, and temporally- and spatially-resolved optical emission.

Voltage and current measurements were performed on the six initial GEC Cells, and a poster paper [2] discussing the measurements was presented at the 1990 GEC (Urbana, IL, Oct. 16–19). Subsequent voltage and current data, along with a description of the GEC Cell design and assembly, were eventually reported in an archival publication [3]. The review paper in this issue by Sobolewski [4] further describes the results and implications of the initial and subsequent comparisons of electrical measurements in detail. Since these initial measurements, a large number of different experiments have been performed using GEC RF Reference Cells, many of which are discussed in this issue. While no orchestrated comparisons of data have been performed in recent years, the large number of operating GEC Cells have now resulted in a significant number of experimental results that are useful to compare and correlate. The results from tests performed on one GEC Cell are of use, not only to modelers, but also in interpreting results from other cells that employ different diagnostics.

## 3. Basic Design and Operation of the GEC Cell

The original design of the GEC RF Reference Cell has been presented in detail in Ref. [3]. A brief description of this design will be presented here, along with information about subsequent changes that have been made to the basic GEC Cell design. We will also briefly discuss some of the special modifications that have been made to GEC Cells by individual groups for various experimental reasons.

**Fig. 1:**
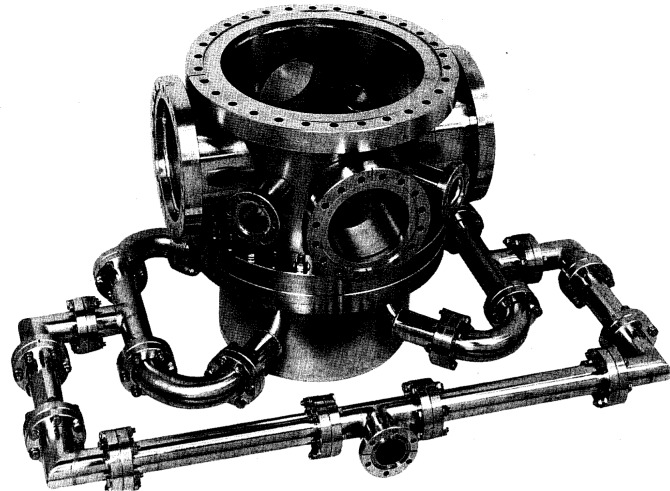
Photograph of the main vacuum chamber of a Gaseous Electronics Conference RF Reference Cell.

Figure 1 shows a photograph of the basic GEC RF Reference Cell vacuum chamber. The main chamber, ports, and manifold are constructed of conventional stainless-steel ultrahigh vacuum (UHV) components. The top and bottom of the chamber consist of $13\frac{1}{4}$ in diameter flanges, and the inner chamber diameter is 25.1 cm. The height of the chamber (as determined by the distance between the faces of the $13\frac{1}{4}$ in flanges) is 22.2 cm.

**Fig. 2:**
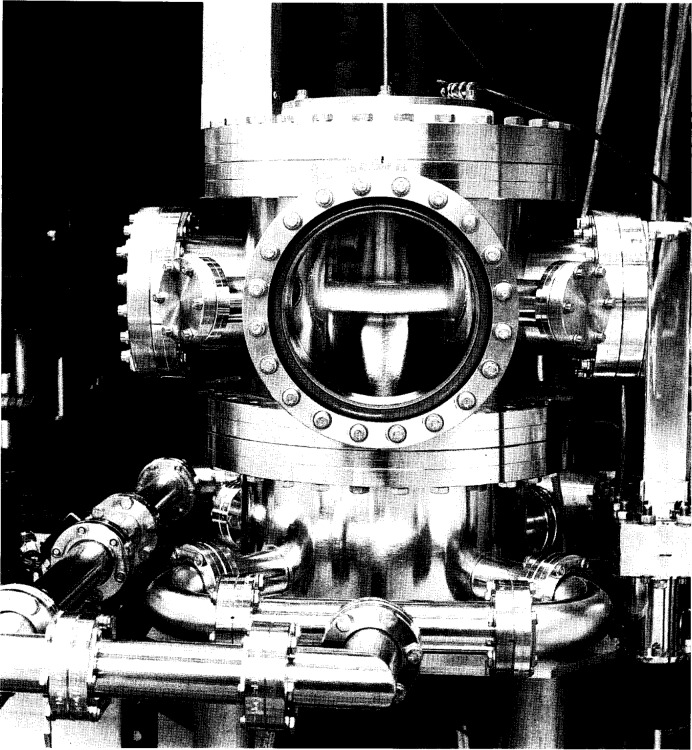
Photograph of a “standard-configuration” GEC Cell sustaining a 200 V, 133 Pa argon plasma.

**Fig. 3:**
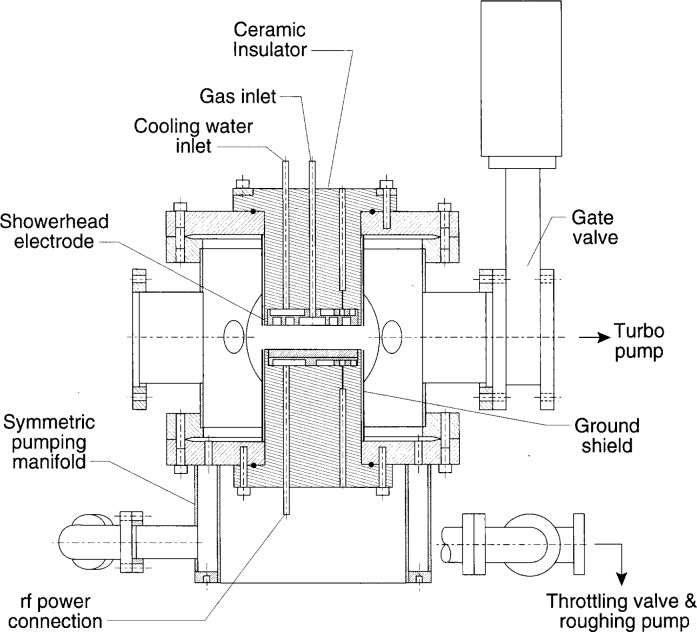
Schematic cross section diagram of a standard-configuration Gaseous Electronics Conference RF Reference Cell.

As can be seen in the photograph in Fig. 2 and the schematic diagram in Fig. 3, a “standard configuration” GEC Cell has two parallel-plate electrodes with a diameter of 10.2 cm (4 in) and a fixed interelectrode spacing of 2.54 cm. These electrodes are supported by ceramic or Teflon^3^ insulators that utilize Viton O-rings for vacuum seals. These insulators provide electrical insulation so each electrode may be powered, grounded, or biased independently. Stainless-steel ground shields surround the insulators and extend from the electrode mounting flange to the plane of the electrode surface. The ground shields reduce sputtering of insulator material, and help to contain the discharge between the electrodes. The electrodes are designed for water cooling, but this feature is often not utilized due to the low power dissipation of many discharges sustained in the GEC Cell.

Optional hardware has been designed for a movable electrode configuration which allows continuous adjustment of the interelectrode spacing from 1.27 cm to 6.35 cm. This option has been installed on only two GEC Cells at this time. Other researchers have varied the electrode spacing by placing aluminum plates on the lower electrode or placing spacers between the insulators and the mounting flanges.

Eight ports are arranged around the mid-plane of the main chamber to provide easy access to the discharge region for diagnostic measurements. Two 8 in ports provide optical access extending beyond the diameter of the electrodes, while four $2\frac{3}{4}$ in ports provide access to the discharge region for pressure gauges, residual gas analyzers, Langmuir probes, and laser beams. Two 6 in ports are also provided, one of which is used for connection to a turbo molecular pump in order to achieve base pressures near 10^−5^ Pa. The distance from the face of the port flanges to the center of the GEC Cell is 19.5 cm for all of the side ports.

Gas is normally supplied to the discharge region of the GEC Cell through holes in the upper electrode, and pumped out via the symmetric pumping manifold (or “octopus”) near the bottom of the GEC Cell. The gas-inlet electrode has 169 equally spaced holes (0.3 mm diameter) placed on concentric circles in a “shower-head” pattern. Normal flow rates used in standard configuration Cells generally range from 1.5 μmol/s to 18.6 μmol/s (2 sccm to 25 sccm). The symmetric pumping manifold, which was designed to reduce angular variation in pumping speed at pressures above 13.3 Pa, restricts the chamber pumping speed when attempting to operate at low pressures. As a consequence, it is generally not possible to achieve pressures below 8 Pa at flow rates greater than 7.5 μmol/s (10 sccm). The conductance of the pumping system is, of course, affected by differences in pumps, foreline traps, valves, and connection hardware. To operate at lower pressures, it is necessary to pump directly with the turbomolecular pump through the 6 in port, rather than through the symmetric pumping manifold. The pressure is controlled either by a variable speed turbo pump, or by a throttling gate valve. The combination of these two pumping techniques allows the GEC Cell to be operated throughout a pressure range of approximately 1 Pa to 133 Pa (approximately 8 mTorr to 1000 mTorr).

The original arrangement for electrical connections to the GEC Cell is discussed in detail in Ref. [3]. Normally, the upper electrode is grounded to the vacuum chamber via a short ground strap, and the rf voltage is applied to the lower electrode through a 0.1 μF capacitor. The current and voltage waveforms are measured by commercial or “homemade” probes, at some point on the copper rod that extends from the back of the lower electrode. To improve the accuracy of the measurements, a shunt circuit [3] is often used to partially cancel the parasitic (or displacement) current that results from the inherent capacitance and inductance of the empty GEC Cell. Measured voltage and current waveforms are analyzed using equivalent circuits, of varying complexity, in order to determine the waveforms at the interface between the plasma and the surface of the powered electrode [3–5]. To achieve agreement between measurements of the higher harmonics of the current and voltage waveforms, it is necessary either to exactly duplicate the external electrical circuitry (including power supply and matching network) [3], or to isolate the external circuitry from the GEC Cell with a low-pass filter [6] that blocks the transmission of the higher harmonics back to the power supply. When using such a filter, the most common connection arrangement is: the rf power supply (with or without a matching network) connected to the low-pass filter, connected with a 1 m cable to a 0.1 μF capacitor in a small box, connected with a 1 m cable to the input to the shunt circuit and the GEC Cell. Initial operating criteria limited the applied peak-to-peak rf voltage to 200 V [3], but in recent years GEC Cells have been operated at applied voltages ranging from 50 V to 1000 V (corresponding to power levels ranging from fractions of a watt to hundreds of watts) without adverse effects.

In an effort to produce a more symmetric discharge, GEC Cells have also been operated in a symmetric, or push-pull, mode, where both electrodes are powered in such a way that the applied voltages are 180° out of phase [7]. This mode is considered easier to model since no dc bias voltage develops on the powered electrode, but it is more complex to set up and operate, and it may be incompatible with some diagnostics.

To date, plasmas have been sustained in GEC Cells in the following gases: Ar, O_2_, N_2_, He, H_2_, SF_6_, CF_4_, C_2_F_6_, CHF_3_, Cl_2_, NF_3_, and various mixtures of these gases. Just as with other plasma reactors, GEC Cells have displayed a sensitivity to past operating history. In particular, changes in the measured electrical characteristics have been observed under some circumstances after the use of chlorine- or fluorine-containing gases, presumably due to changes in the condition of the surfaces of the chamber and the electrodes. The magnitudes of these changes vary. Often the long term effects of these changes can be removed by running discharges in other gases, such as pure argon or oxygen, or by polishing the electrodes.

While, under most conditions of interest, the plasmas generated in the GEC Cell behave in a stable manner, there have been instances where unstable discharge behavior has been observed, manifested, for example, by time-dependent nonstationary behavior of the optical emission. The most common instability occurs in argon plasmas at high pressures and voltages (near 133 Pa and 200 V), and manifests itself as localized regions of increased emission (“glowing spheres”) between the parallel plates that rotate about the central axis of the electrodes. This instability occurs primarily in new GEC Cells, and has been observed to occur less frequently the longer the GEC Cell has been in operation. Other instabilities have occasionally been observed for different gas mixtures and operating conditions, but these instabilities tend to be unique to individual GEC Cells and are dependent upon past usage.

Over the years, several “official” changes (agreed upon by an ad hoc users’ group) have been made to the design of the GEC RF Reference Cell. The most significant involves modifications to the design of the insulators that support the electrodes. The original insulators were nearly solid and were fabricated from 99.5 % alumina (Al_3_O_2_). Difficulties in fabricating these insulators resulted in some GEC Cells being assembled with insulators that were machined from Teflon stock. Some time later, the ceramic insulators were redesigned with a hollow center (which made them easier to cast), and were specified to be fabricated from 95 % alumina (which is less susceptible to chipping). The capacitance and inductance of the GEC Cell are affected by the different insulator designs and materials, and this must be taken into account when analyzing the measured electrical waveforms [3–5]. The ceramic insulators are quite expensive and suppliers continue to be difficult to find. Thus many of the newest GEC Cells have been equipped with Teflon insulators that are relatively inexpensive and easy to fabricate. There has been no evidence of adverse performance due to long term usage of Teflon insulators in the GEC Cell, provided the ground shields are used.

Other less dramatic changes in the “official” Cell design include the addition of two small bellows to the symmetric pumping manifold to simplify fabrication, switching to a “captured” O-ring groove to seal the lower insulator to the chamber, using larger O-rings to improve the seal between the electrodes and the insulators, and numerous minor modifications to the optional, moveable electrode assembly. Mechanical drawings for the GEC Cell, including the most recent modifications, are available on computer diskette.^4^

Unofficial changes to GEC Cells, i.e., changes made by individual groups for specific research requirements, are also numerous, and many of them are discussed in the subsequent articles in this Special Issue, along with their influence on GEC Cell performance. Some of the modifications include the use of stainless-steel electrodes instead of aluminum [8, 9], the installation of load locks [10], redesign of the grounded electrode for mass spectrometric sampling [9], operating the Cell with no upper electrode [11], and the installation of an insulating ring around the powered electrode to trap dust particles [11].

The basic design of the GEC Cell has recently been extended to allow the installation of an inductively-coupled plasma (ICP) source in place of the standard upper electrode assembly [12]. This source is similar in design to industrial inductively-coupled reactors, and was originally designed to validate models of inductively-coupled etching plasmas. At present, there are approximately five GEC Cells that have been assembled or modified to use the new ICP source, and intercomparison of results is forthcoming. Design drawings of the ICP source are also available on diskette.^4^

## 4. Significant Results

When the concept of a reference cell was first discussed at the 1988 GEC, it was met with some skepticism. There was much debate over whether a suitable reference cell system could be developed and whether such a system would be productive. In addition, there were many different opinions as to what a reference cell should look like. The fact that the “final” GEC RF Reference Cell design was a dramatic compromise between an industrial reactor, suitable for process development, and a simple symmetric system, suitable for modeling, suggested to many that the GEC Cell would be useful for neither. Furthermore, persuasive arguments were presented that parallel-plate technology would soon be discarded and that development of a reference cell based on this geometry would not be useful. In hindsight, it is evident that GEC Cell studies, based on a parallel-plate design, have yielded valuable results. The GEC Cell has proven to be well suited for plasma research due to its combination of simplicity and flexibility for diagnostic application. Furthermore, many of the results of GEC Cell investigations are system independent, having relevance for general rf discharge studies.

As discussed previously, much of the initial work with the GEC Cells was devoted to determining whether rf discharges created in different GEC Cells would have similar characteristics for similar input conditions (pressure, power, etc.). This was an important issue for meaningful scientific investigation and for comparison of results from different laboratories. In addition, it was an important technological issue; for many years, users of commercial plasma-etching equipment had observed that supposedly identical plasma-etching chambers displayed different etching characteristics. Studies with the GEC Cell showed that “identical” etching chambers could be made to operate in a similar manner, e.g., similar electrical characteristics, electron densities, and etching rates.

One of the important issues affecting chamber-to-chamber reproducibility turned out to be the external rf drive circuitry. GEC Cell studies clearly showed that, due to the nonlinear nature of plasmas, it is necessary to pay a great deal of attention to the external rf drive circuitry in order to create “identical” plasmas in different systems. The understanding of external circuit effects, derived from the GEC Cell studies, was used not only to obtain similar operation in different GEC Cells, but also to examine and eliminate chamber-to-chamber variability in commercial plasma etching reactors [13]. Methods of isolating external circuitry from the plasma were also developed [6].

Another outcome of the GEC Cell studies was the development and evaluation of rf measurement techniques. In principle, voltage and current measurements are straightforward. In practice, there can be many difficulties associated with shielding and grounding, and with the accurate measurement of phase (as well as magnitude) at frequencies from 13.56 MHz to 67.80 MHz (5th harmonic) [4, 5]. Researchers working with GEC Cells found practical, effective solutions for these problems, which were disseminated to the scientific community and to commercial industry. The process of carefully comparing electrical measurements from different GEC Cells also showed the importance of detailed analysis of the measured electrical waveforms using the electrical characteristics of the reactor in order to calculate the power dissipated in the plasma. Comparison of measurements on GEC Cells showed significant discrepancies between power readings on rf power supplies and calculated power dissipation, implying that power levels stated in much of the rf plasma literature may be of little use when comparing results or characterizing a discharge.

One of the most significant contributions from use of the GEC Cell is the large amount of experimental data from many diagnostics that has been accumulated under similar plasma conditions. For example, electron densities, electric-field strengths, ion-energy distributions, and metastable densities have all been measured for plasmas sustained in helium. For argon, the list presently includes electron densities, ion-energy distributions, metastable densities, electron-energy distribution functions, and temporally- and spatially-resolved optical emission. The lists of measurements applied to plasmas generated in other gases are shorter, but are constantly being added to. These data represent a “basic data set” that begins to fully characterize a discharge so as to promote and validate plasma modeling.

Use of the GEC Cell has stimulated the plasma modeling community. As seen in the two review articles contained in this Special Issue of the *Journal of Research of the National Institute of Standards and Technology* [14, 15], significant progress has been made in the modeling of various discharges in the GEC Cell, due in part to the availability of experimental data for comparison. Additionally, the emergence of the reference-cell concept prompted the modeling community to initiate a “benchmark model comparison,” where each modeler applied their own model to a given set of experimental conditions [16]. The agreed-upon “computational reference cell” that formed the basis of this comparison was simpler than the GEC RF Reference Cell, but the exercise was very useful for determining the validity of many models over a range of plasma conditions.

The measurement of particular plasma parameters on different Cells using different diagnostics has also been shown to be useful. For example, electron densities have now been measured for similar plasma conditions in four different GEC Cells [8] using Langmuir probes, microwave interferometry, and laser-induced fluorescence. Comparison of results from measurements using different techniques not only provides validation of these techniques, but also provides a measure of the reliability and reproducibility of the experimental measurements and of the discharges themselves.

## 5. Concluding Comments

Researchers using the GEC RF Reference Cell have successfully addressed problems in low temperature plasma science of interest to industry, experimentalists, and modelers. Much of the work done was facilitated by the “reference-cell” concept. This concept has evolved in response to the technical needs of the plasma community. The introduction of “push-pull” excitation of the electrodes is one example, as is the development of an inductively-coupled plasma source. Other changes in the GEC Cell configuration, such as the attachment of load locks, have also kept the GEC Cell “relevant” to industrial etching processes. Future plans include the development of an entirely new GEC Cell design that would more closely emulate the design of industrial reactors, while maintaining the diagnostic access necessary for plasma research. These plans should continue to make the reference-cell concept a valuable tool for basic understanding of rf plasmas, and for advancement and improvement of industrial plasma processing.

## Footnotes

1 Sandia National Laboratories, National Institute of Standards and Technology, Wright Laboratories, University of New Mexico, AT&T Bell Labs, and the University of Michigan.

2 The occurrence of the 1989 earthquake in the San Francisco Bay area abruptly ended this workshop, and sent all the participants scurrying for the parking lot before a consensus could be reached.

3 Certain commercial equipment, instruments, or materials are identified in this paper to specify adequately the experimental procedure. Such identification does not imply recommendation or endorsement by the National Institute of Standards and Technology, nor does it imply that the materials or equipment identified are necessarily the best available for the purpose.

4 For information contact: James K. Olthoff, NIST, B344/220, Gaithersburg, MD 20899 or via e-mail (olthoff@eeel.nist.gov).

## 7. Appendix A. Bibliography

This appendix contains an up-to-date bibliography of all archival and conference publications and reports that present results of research performed on GEC RF Reference Cells as of July 1995. Only papers and reports that are considered referenceable are listed here, so presentations from conferences that do not publish a significant proceedings are not included (such as presentations at the Gaseous Electronics Conference). Papers that are “in press” or have been submitted to a journal are included for completeness. The listing is in approximate order of publication, and has been split into groups of papers that contain either primarily experimental or theoretical results.

### Experimental

1. Electrical characterization of rf plasma discharges
  P. A. Miller and M. Kamon, SETEC Report 90-0009, 1990.
2. The GEC RF Reference Cell: Diagnostic techniques and initial results
  K. E. Greenberg, P. J. Hargis, and P. A. Miller, SETEC Report 90-013, 1990.
3. Measurements on the NIST GEC Reference Cell
  J. R. Roberts, J. K. Olthoff, R. J. Van Brunt, and J. R. Whetstone, in Advanced Techniques for Integrated Circuit Processing, (Society of Photo-Optical Instrumentation Engineers, SPIE, 1990), Vol. 1392, p. 428.
4. Status of the GEC Reference Cell / Laser diagnostics of plasma etching discharges
  P. J. Hargis, Jr., K. E. Greenberg, and P. A. Miller, Intl. Seminar of Reactive Plasmas, Nagoya, Japan (17–19 June 1991).
5. Mass spectrometric and optical emission diagnostics for rf plasma reactors
  J. K. Olthoff, J. R. Roberts, R. J. Van Brunt, J. R. Whetstone, M. A. Sobolewski, and S. Djurovic, in Process Module Metrology, Control, and Clustering, (Society of Photo-Optical Instrumentation Engineers, SPIE, 1991), Vol. 1595, p. 168.
6. Electrical characterization of rf plasmas
  P. A. Miller, in Process Module Metrology, Control, and Clustering, (Society of Photo-Optical Instrumentation Engineers, SPIE, 1991), Vol. 1595, p. 179.
7. Application of chemometrics to optical emission spectroscopy for plasma monitoring
  M. P. Splichal and H. M. Anderson, in Process Module Metrology, Control, and Clustering, (Society of Photo-Optical Instrumentation Engineers, SPIE, 1991), Vol. 1595, p. 189.
8. Electrical isolation of radio-frequency plasma discharges
  P. A. Miller, H. A. Anderson, and M. P. Splichal, *J. Appl. Phys*. 71, 1171 (1992).
9. Period-doubling bifurcation in a plasma reactor
  P. A. Miller and K. E. Greenberg, *Appl. Phys. Lett*. 60, 2859 (1992).
10. Diagnostic measurements in rf plasmas for materials processing J. R. Roberts, J. K. Olthoff, M. A. Sobolewski, R. J. Van Brunt, J. R. Whetstone, and S. Djurovic, in Atomic Processes in Plasmas, AIP Conference Proceedings 257, (American Institute of Physics, New York, 1992), p. 157.
11. Ion kinetic-energy distributions and electrical measurements in argon-oxygen rf glow discharges
  J. K. Olthoff, R. J. Van Brunt, and M. A. Sobolewski, in Proc. Tenth Intl. Conf. on Gas Discharges and Their Applications, (University College of Swansea, Swansea, Wales, U. K.1992), p. 440.
12. Ion kinetic-energy distributions in rf glow discharges
  J. K. Olthoff, R. J. Van Brunt, and S. B. Radovanov, *J. Appl. Phys*. 72, 4566 (1992).
13. Electrical characterization of radio-frequency discharges in the Gaseous Electronics Conference Reference Cell
  M. A. Sobolewski, *J. Vac. Sci. Technol. A* 10, 3550 (1992).
14. Measurements and analysis of the equivalent circuit of the GEC RF Reference Cell
  J. T. Verdeyen, Sandia Report SAND92-7284, 1992.
15. Electrical measurements for monitoring and control of rf plasma processing
  M. A. Sobolewski and J. R. Whetstone, in Advanced Techniques for Integrated Circuit Processing II, (Society of Photo-Optical Instrumentation Engineers, SPIE, 1992), Vol. 1803, p. 309.
16. Absolute spatially- and temporally-resolved optical emission measurements of rf glow discharges in argon
  S. Djurovic, J. R. Roberts, M. A. Sobolewski, and J. K. Olthoff, *J. Res. Natl. Inst. Stand. Technol*. 98, 159 (1993).
17. Electron and metastable densities in parallel-plate radio-frequency discharges
  K. E. Greenberg and G. A. Hebner, *J. Appl. Phys*. 73, 8126 (1993).
18. Radial optical emission profiles of radio-frequency glow discharges
  J. Pender, M. Buie, T. Vincent, J. Holloway, M. Elta, and M. Brake, *J. Appl. Phys*. 74, 3590 (1993).
19. Subharmonics and RF-plasma sheaths
  P. A. Miller, L. A. Romero, and P. D. Pochan, *Phys. Rev. Lett*. 71, 863 (1993).
20. Hydrogen Balmer Alpha line shapes for hydrogen-argon mixtures in a low pressure rf discharge
  S. Djurovic and J. R. Roberts, *J. Appl. Phys*. 74, 6558 (1993).
21. A comparison of electron density measurements made using a Langmuir probe and microwave interferometer in the Gaseous Electronics Conference reference reactor
  L. J. Overzet and M. B. Hopkins, *J. Appl. Phys*. 74, 4323 (1993).
22. Spatial variations in the charge density of argon discharges in the Gaseous Electronics Conference reference reactor
  M. B. Hopkins, L. J. Overzet, and M. Turner, *Appl. Phys. Lett*. 63, 2484 (1993).
23. Electric-field measurements in 13.56-MHz helium discharges
  K. E. Greenberg and G. A. Hebner, *Appl. Phys. Lett*. 63, 3282 (1993).
24. Electrical sensors for monitoring rf plasma sheaths
  M. A. Sobolewski and J. K. Olthoff, in Microelectronic Processes, Sensors and Controls, (Society of Photo-Optical Instrumentation Engineers, SPIE, 1993), Vol. 2091, p. 290.
25. Optical emission spectroscopy on the GEC reference cell
  M. J. Buie, J. T. Pender, T. Vincent, J. Holloway, M. Brake, and M. Elta, in Microelectronic Processes, Sensors and Controls, (Society of Photo-Optical Instrumentation Engineers, SPIE, 1993), Vol. 2091, p. 211.
26. An integrated system of optical sensors for plasma monitoring and plasma process control
  H. M. Anderson and M. P. Splichal, in Microelectronic Processes, Sensors and Controls, (Society of Photo-Optical Instrumentation Engineers, SPIE, 1993), Vol. 2091, p. 333.
27. Plasma-etching science meets technology in the MDL
  K. E. Greenberg, P. A. Miller, R. Patteston, and B. K. Smith, Sandia National Laboratories Report, SAND93-0187, March 1993.
28. Plasma standard cell for laboratory demonstrations and experiments
  R. L. Lane and T. J. Grimsley, Tenth Biennial University/Government/Industry Microelectronics Symposium, (IEEE, Piscataway, NJ, 1993), p. 213.
29. Kinetic-energy distributions of ions sampled from argon plasmas in a parallel-plate rf reference cell
  J. K. Olthoff, R. J. Van Brunt, S. B. Radovanov, J. A. Rees, and R. Surowiec, *J. Appl. Phys*. 75, 115 (1994).
30. The GEC RF Reference Cell: A parallel-plate radio frequency system to study plasma-processing discharges
  P. J. Hargis, Jr., K. E. Greenberg, P. A. Miller, J. B. Gerardo, J. R. Torczynski, M. E. Riley, G. A. Hebner, J. R. Roberts, J. K. Olthoff, J. R. Whetstone, R. J. Van Brunt, M. A. Sobolewski, H. M. Anderson, M. P. Splichal, J. L. Mock, P. Bletzinger, A. Garscadden, R. A. Gottscho, G. Selwyn, M. Dalvie, J. E. Heidenreich, J. W. Butterbaugh, M. L. Brake, M. L. Passow, J. Pender, A. Lujan, M. E. Elta, D. B. Graves, H. H. Sawin, M. J. Kushner, J. T. Verdeyen, R. Horwath, and T. R. Turner, *Rev. Sci. Instrum*. 65, 140 (1994).
31. Use of an ion energy analyzer-mass spectrometer to measure ion kinetic-energy distributions from rf discharges in argon-helium gas mixtures
  J. K. Olthoff, R. J. Van Brunt, S. B. Radovanov, and J. A. Rees, *IEE Proc.—Sci. Meas. Technol*. 141, 105 (1994).
32. Particulates in C_2_F_6_-CHF_3_ and CF_4_-CHF_3_ etching plasmas
  H. M. Anderson, S. B. Radovanov, J. L. Mock, and P. J. Resnick, *Plasma Sources Sci. Technol*. 3, 302 (1994).
33. The effect of plasma-surface interactions on the radial variation of H atom density in a hydrogen RF discharge
  B. N. Ganguly and P. Bletzinger, *J. Appl. Phys*. 76, 1476 (1994).
34. Electric fields in high-frequency parallel-plate helium discharges
  G. A. Hebner, K. E. Greenberg, and M. E. Riley, *J. Appl. Phys*. 76, 4036 (1994).
35. The effects of electrostatic, molecular drag and gravitational forces on the behavior of particle clouds in an rf discharge
  J. F. O’Hanlon, J. Kang, L. K. Russell, and L. Hong, *IEEE Trans. Plasma Sci*. 22, 122 (1994).
36. Detection and modelling of electrode topography effects on particle traps
  M. Dalvie, M. Surrendra, G. S. Selwyn, and C. R. Guarnieri, *Plasma Sources Sci. Technol*. 3, 442 (1994)
37. Two-dimensional argon metastable density measurements in an rf glow discharge by planar laser-induced fluorescence imaging
  B. K. McMillin and M. R. Zachariah, *J. Appl. Phys*. 77, 5538 (1995).
38. Time-resolved Balmer-alpha emission from fast hydrogen atoms in low pressure, radio-frequency discharges in hydrogen
  S. B. Radovanov, K. Dzierzega, J. R. Roberts, and J. K. Olthoff, *Appl. Phys. Lett*. 66, 2637 (1995).
39. In situ diode laser absorption measurements of plasma species in a GEC reference cell reactor
  D. B. Oh, A. C. Stanton, H. M. Anderson, and M. P. Splichal, *J. Vac. Sci. Technol. B* 13, 954 (1995).
40. The Gaseous Electronics Conference RF Reference Cell—An Introduction
  J. K. Olthoff and K. E. Greenberg, *J. Res. Natl. Inst. Stand. Technol*. 100, 327 (1995).
41. Current and voltage measurements in the Gaseous Electronics Conference RF Reference Cell
  M. A. Sobolewski, *J. Res. Natl. Inst. Stand. Technol*. 100, 341 (1995).
42. Optical emission on the Gaseous Electronics Conference RF Reference Cell
  J. R. Roberts, *J. Res. Natl. Inst. Stand. Technol*. 100, 353 (1995).
43. Optical diagnostics in the Gaseous Electronics Conference RF Reference Cell
  G. A. Hebner and K. E. Greenberg, *J. Res. Natl. Inst. Stand. Technol*. 100, 373 (1995).
44. Studies of ion kinetic-energy distributions in the Gaseous Electronics Conference RF Reference Cell
  J. K. Olthoff, R. J. Van Brunt, and S. B. Radovanov, *J. Res. Natl. Inst. Stand. Technol*. 100, 383 (1995).
45. Microwave diagnostics in the Gaseous Electronics Conference RF Reference Cell
  L. J. Overzet, *J. Res. Natl. Inst. Stand. Technol*. 100, 401 (1995).
46. Langmuir probe measurements in the Gaseous Electronics Conference RF Reference Cell
  M. B. Hopkins, *J. Res. Natl. Inst. Stand. Technol*. 100, 415 (1995).
47. An inductively coupled plasma source for the Gaseous Electronics Conference RF Reference Cell
  P. A. Miller, G. A. Hebner, K. E. Greenberg, P. D. Pochan, and B. P. Aragon, *J. Res. Natl. Inst. Stand. Technol*. 100, 427 (1995).
48. Reactive ion etching in the Gaseous Electronics Conference RF Reference Cell
  M. L. Brake, J. T. P. Pender, M. J. Buie, A. Ricci, J. Soniker, P. D. Pochan, and P. A. Miller, *J. Res. Natl. Inst. Stand. Technol*. 100, 441 (1995).
49. Dusty plasma studies in the Gaseous Electronics Conference RF Reference Cell
  H. M. Anderson and S. B. Radovanov, *J. Res. Natl. Inst. Stand. Technol*. 100, 449 (1995).
50. Surface roughness studies of deep plasma etching in crystalline silicon
  R. L. Lane and Z. Li, in Proc. 11th Biennial University-Government-Industry-Microelectronics Symposium (SEMATECH, Austin, TX, 1995), p. 134.
51. Ion energy distributions and Balmer-Alpha excitation in Ar-H_2_ radio frequency discharges
  S. B. Radovanov, J. K. Olthoff, R. J. Van Brunt, and S. Djurovic, *J. Appl. Phys*. 78, 746 (1995).
52. Effect of electrode material on measured ion energy distributions in radio-frequency discharges
  J. K. Olthoff, R. J. Van Brunt, and S. B. Radovanov, *Appl. Phys. Lett*. 67, 473 (1995).
53. Kinetic-energy distributions of ions sampled from radio-frequency discharges in helium, nitrogen, and oxygen
  R. J. Van Brunt, J. K. Olthoff, and S. B. Radovanov, in Proc. Eleventh Intl. Conf. on Gas Discharges and Their Applications, (Tokyo, Japan, 1995), in press.
54. Influence of electrode material on measured ion kinetic-energy distributions in radio-frequency discharges
  R. J. Van Brunt, J. K. Olthoff, and S. B. Radovanov, in Proc. International Conference on the Physics of Ionized Gases, (Stevens Institute of Technology, Hoboken, NJ, 1995), in press.
55. 2-D imaging of CF_2_ density by laser-induced fluorescence of CF_4_ etching plasmas in the GEC RF Reference Cell
  B. K. McMillin and M. R. Zachariah, in Proc. 12th International Symposium on Plasma Chemistry, (Minneapolis, MN, 1995), in press.
56. In situ diagnostics for etch uniformity
  M. Buie, J. Pender, M. Elta, and M. Brake, *J. Vac. Sci. Technol*. A, in press (Aug. 1995).
57. Time resolved power measurements to pulsed discharges in the Gaseous Electronics Conference reference reactor
  L. J. Overzet and F. Y. Leong-Rousey, *Plasma Sources Sci. Technol*., in press (July/August).
58. Electrical characteristics of argon radio-frequency glow discharges in an asymmetric cell
  M. A. Sobolewski, IEEE Trans. Plasma Sci., in press.
59. 2-D laser-induced fluorescence imaging of metastable density in low-pressure rf argon plasmas with added O_2_, Cl_2_, or CF_4_
  B. K. McMillin and M. R. Zachariah, *J. Appl. Phys*., in press.
60. 2-D images of CF_2_ density in CF_4_/Ar plasmas by laser-induced fluorescence in a GEC RF Reference Cell
  B. K. McMillin and M. R. Zachariah, IEEE Trans. Plasma Sci., submitted.
61. 2-D images of argon metastable density in rf plasmas by laser-induced fluorescence in a GEC Reference Cell
  B. K. McMillin and M. R. Zachariah, *IEEE Trans. Plasma Sci*., submitted.
62. Abels inversion applied to experimental spectroscopic data
  M. J. Buie, J. T. P. Pender, J. Holloway, T. Vincent, P. Ventzek, and M. Brake, *J. Quant. Rad. Spectroscopy*, submitted.
63. In situ sensor of spatially resolved optical emission
  M. J. Buie, J. T. P. Pender, J. Holloway, and M. Brake, *IEEE Trans. Plasma Sci*., submitted.
64. Comparison of Si etching in the GEC reference cell and a commercial reactive ion etcher
  J. Pender, M. Buie, and M. L. Brake, submitted.
65. Sheath phenomena in rf driven argon and hydrogen plasmas observed by two dimensional imaging of optical emission
  C. M. O. Mahony, R. C. Cheshire, R. Al-Wazzan, W. G. Graham, *Appl. Phys. Lett*., submitted.
66. Electron density measurements in an rf helium discharge by laser induced fluorescence method
  K. Dzierzega and K. Musiol, *J. Appl Phys*., submitted.

### Modeling

1. Numerical investigation of the kinetics and chemistry of rf glow discharge plasmas sustained in He, N_2_, O_2_, He/N_2_/O_2_, He/CF_4_/O_2_, and SiH_4_/NH_3_ using a Monte Carlo-fluid hybrid model
  T. J. Sommerer and M. J. Kushner, *J. Appl. Phys*. 71, 1654 (1992).
2. Monte Carlo-fluid model of chlorine atom production in Cl_2_, HCl, and CCl_4_ radio-frequency discharges for plasma etching. T. J. Sommerer and M. J. Kushner, *J. Vac. Sci. Technol. B* 10, 2179 (1992).
3. Radio frequency discharge modeling: Moment equations approach
  M. Meyyappan and T. R. Govindan, *J. Appl. Phys*. 74, 2250 (1993).
4. Fluid simulations of glow discharges: Effect of metastable atoms in argon
  D. P. Lymberopoulos and D. J. Economou, J. Appl. Phys. 73, 3668 (1993).
5. Fluid simulations of radio frequency glow discharges: Two-dimensional argon discharge including metastables
  D. P. Lymberopoulos and D. J. Economou, *Appl. Phys. Lett*. 63, 2478 (1993).
6. A comparative study between non-equilibrium and equilibrium models of rf glow discharges
  F. F. Young and C. H. Wu, *J. Phys. D: Appl. Phys*. 26, 782 (1993).
7. Theoretical and experimental study of low-temperature, capacitively-coupled, RF-driven helium plasmas
  M. E. Riley, K. E. Greenberg, G. A. Hebner, and P. J. Drallos, *J. Appl. Phys*. 75, 2789 (1994).
8. Modeling and simulation of glow discharge plasma reactors
  D. P. Lymberopoulos and D. J. Economou, *J. Vac. Sci. A* 12, 1229 (1994).
9. Investigation of electron source and ion flux uniformity in high plasma density inductively coupled etching tools using two-dimensional modeling
  P. L. Ventzek, M. Grapperhaus, and M. S. Kushner, *J. Vac. Sci. Technol. B* 12, 3118 (1994).
10. D model of a capacitively coupled rf discharge and comparisons with experiments in the GEC reference reactor
  J. P. Boeuf and L. C. Pitchford, *Phys. Rev. E* 51, 1376 (1995).
11. Radio frequency voltage division between two plasma sheaths in the Gaseous Electronics Conference reference cell
  Y. Wang, *Appl. Phys. Lett*. 66, 2186 (1995).
12. Spatiotemporal electron dynamics in radio-frequency glow discharges: fluid versus dynamic Monte Carlo simulations
  D. P. Lymberopoulos and D. J. Economou, *J. Phys. D: Appl. Phys*. 28, 727 (1995).
13. One-dimensional modeling studies of the Gaseous Electronics Conference RF Reference Cell
  T. R. Govindan and M. Meyyappan, *J. Res. Natl. Inst. Stand. Technol*. 100, 463 (1995).
14. Two-dimensional self-consistent radio-frequency plasma simulations relevant to the Gaseous Electronics Conference RF Reference Cell
  D. P. Lymberpopoulos and D. J. Economou, *J. Res. Natl. Inst. Stand. Technol*. 100, 473 (1995).
15. Modeling and simulation of two-dimensional reactive plasma flow in inductively coupled reactors
  D. P. Lymberpopoulos, R. Wise, and D. J. Economou, Proceedings of the First Symposium on Process Control, Diagnostics, and Modeling in Semiconductor Manufacturing, (The Electrochemical Society, Spring meeting, Reno, NV, 1995), in press.
16. Evidence for inelastic processes for N_3_^+^and N_4_^+^ from ion energy distributions in He/N_2_ rf glow discharges
  H. H. Hwang, J. K. Olthoff, R. J. Van Brunt, S. B. Radovanov, and M. J. Kushner, J. Appl. Phys., submitted.
17. Collision-mediated stochastic heating of the electrons in an rf sheath
  Y. Wang, Appl. Phys. Lett., submitted.
